# Occult Tethered Cord Syndrome: Clinical Characteristics, Diagnostic Challenges, and Management Considerations

**DOI:** 10.7759/cureus.98747

**Published:** 2025-12-08

**Authors:** Michihiro Kurimoto

**Affiliations:** 1 Pediatric Neurosurgery, Aichi Children's Health and Medical Center, Ōbu, JPN

**Keywords:** filum terminale, occult tethered cord syndrome, pediatric neurosurgery, spinal dysraphism, surgical outcomes, urodynamics

## Abstract

Occult tethered cord syndrome (OTCS) is defined as the clinical manifestation of a tethered cord without radiographic evidence of a low-lying conus; however, there is no consensus on surgical indications or evaluation criteria. We present three cases treated surgically at our hospital, and in contrast to that, a narrative review of the literature on OTCS was conducted, focusing on epidemiology, pathophysiology, clinical features, diagnostic tools, and surgical versus conservative management. Emphasis was placed on pediatric populations, in whom the syndrome is most frequently described.

Between April 2010 and June 2025, three patients with OTCS underwent surgical sectioning of the filum terminale at our institution. All patients presented with urological abnormalities, and one patient also demonstrated orthopedic involvement. Improvement in urological symptoms was observed in two patients (66%), including resolution of vesicoureteral reflux and nocturnal enuresis, whereas no improvement was noted in orthopedic abnormalities. No perioperative complications were observed. During the follow-up period (median (interquartile range) = 1281 (1264-1490) days), these results indicated that while urinary symptoms after surgical dislocation were highly likely to recover, orthopedic symptoms remained largely refractory in the majority of cases. OTCS remains controversial and heterogeneous. Diagnosis depends largely on clinical judgment, and treatment decisions must balance the potential surgical benefits with the risks and uncertain long-term outcomes. Prospective controlled studies and the development of objective biomarkers are essential for establishing standardized diagnostic and therapeutic guidelines.

## Introduction

Occult tethered cord syndrome (OTCS) is defined as a condition in which patients present with symptoms such as urinary dysfunction or lower-extremity motor abnormalities, despite normal findings on spinal imaging, including magnetic resonance imaging (MRI). Common radiological abnormalities associated with classic tethered cord syndrome (TCS) include spinal lipomas, a low-lying conus medullaris, and a thickened filum terminale; however, OTCS represents a rare entity in which neurological deficits occur in the absence of these abnormalities. Problems associated with OTCS include an uncertain pathological basis, and there are no overt imaging findings; surgical indications must rely on clinical symptoms and functional testing (e.g., urodynamic studies), for which no standardized evaluation criteria exist. Although symptom improvement after surgery has been reported, outcomes such as improvement and recurrence rates vary widely across studies, and the therapeutic efficacy remains uncertain. Here, we review pediatric OTCS cases treated at our institution and discuss the diagnostic and therapeutic considerations, considering the literature.

The conceptual origin of OTCS can be traced to the mid-20th century, when the broad clinical recognition of TCS emerged. Hoffman et al. clarified the clinical features of TCS associated with spina bifida and radiological abnormalities [1]. Early surgical intervention resulted in symptomatic improvement, confirming the pathological significance of spinal cord tethering. However, subsequent clinical observations identified groups of pediatric patients often presenting with neurogenic bladder dysfunction or orthopedic manifestations that exhibited symptoms consistent with TCS but did not fulfill the conventional radiological diagnostic criteria. This discrepancy led to the proposal of “occult” or “imaging-negative” TCS, which was first systematically described in the 2000s [2,3]. The introduction of urodynamic testing and advanced electrophysiological evaluation further supports the possibility that subtle spinal traction can impair neural function even in the absence of demonstrable imaging abnormalities.

## Case presentation

At our facility, surgical procedures were performed on three patients with OTCS between April 2010 and June 2025. All three patients presented with urological abnormalities, and one patient presented with orthopedic abnormalities. The median age at surgery was 88 months (range 11-125 months), with two female patients and one male patient.

**Figure 1:**
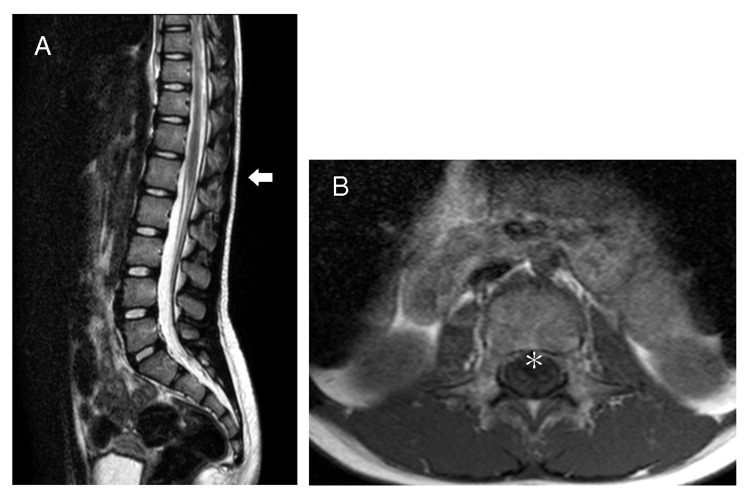
Case 1: A: T2 sagittal image showing the level of the conus medullaris. B: T1 axial image just caudal to the conus medullaris. No low conus is observed (white arrow), and no attachments, including a spinal lipoma, are detected (asterisk).

Case 1 was referred to our hospital with nocturnal enuresis and limited bilateral ankle dorsiflexion at 120 months of age. Spinal MRI showed that the conus medullaris was at the L1/2 level, with no evidence of spinal lipoma or tethering (Figure 1), and preoperative urodynamic studies were unremarkable. To regard nocturnal enuresis as symptomatic, at 125 months of age, a filum terminal section was obtained via a laminotomy approach. No perioperative complications were observed. During the six-month follow-up period, nocturnal enuresis improved; however, there was no change in ankle dorsiflexion limitation.

**Figure 2:**
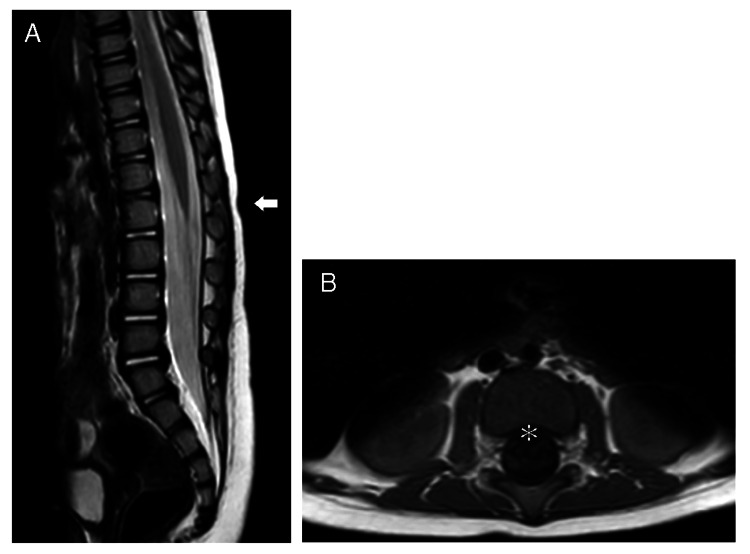
Case 2: A: T2 sagittal image showing the level of the conus medullaris. B: T1 axial image just caudal to the conus medullaris. No low conus is observed (white arrow), and no attachments, including a spinal lipoma, are detected (asterisk).

Case 2 was referred to our hospital after a urodynamic study revealed early overactive bladder at 26 months of age. Spinal MRI showed the conus medullaris at the L1/2 level, with no evidence of spinal lipoma or tethering (Figure 2). This patient had a history of an intermediate imperforate anus and complete transposition of the great arteries. At 32 months of age, a filum terminal section was performed using the interlaminar approach, and no perioperative complications were observed. During the three-year follow-up period, no improvement was observed in the early overactive bladder. The patient has a history of intermediate imperforate anus and complete transposition of the great arteries, but both conditions were assessed as unrelated to the residual symptoms.

**Figure 3:**
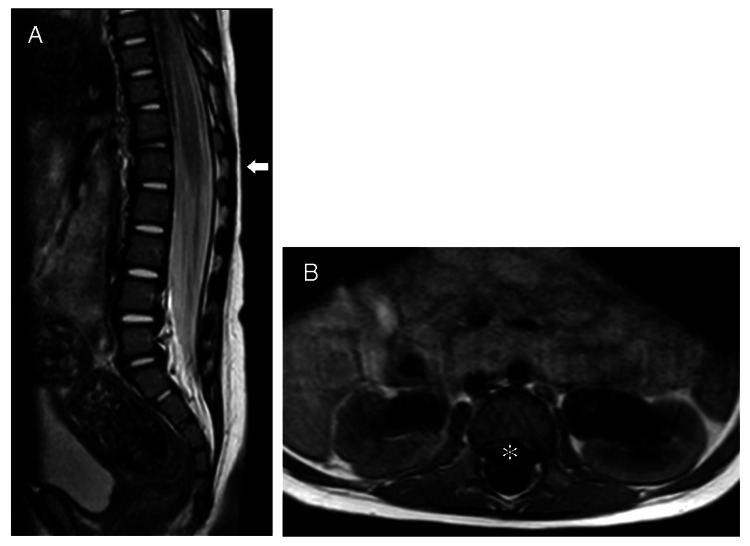
Case 3: A: T2 sagittal image showing the level of the conus medullaris. B: T1 axial image just caudal to the conus medullaris. No low conus is observed (white arrow), and no attachments, including a spinal lipoma, are detected (asterisk). T2 sagittal images showing the level of the conus medullaris and T1 axial images just caudal to the conus medullaris are shown. No low conus is observed (white arrow), and no attachments, including a spinal lipoma, are detected (asterisk).

Case 3 was referred to our hospital for further examination at nine months of age because of bilateral vesicoureteral reflux and bladder wall irregularities. Spinal MRI showed the conus medullaris at the L2 level, with no evidence of spinal lipoma or tethering (Figure 3). At 11 months of age, a filum terminal section was performed using the interlaminar approach, and no perioperative complications were observed. At the ninth-month postoperative urological evaluation, vesicoureteral reflux was confirmed to have resolved and had not recurred.

Of the three patients, improvement in urological abnormalities was observed in two patients, whereas no improvement was noted in orthopedic abnormalities (Table 1).

**Table 1:** Predictor variables and outcomes of three occult tethered cord syndrome children Data are presented as the unweighted number (percentage) of patients unless otherwise indicated

Variables (n = 3)	Overall
Age at surgery (months), median	88 (11-125)
Female sex	2
Preoperative abnormal findings in Urology	
Nocturnal enuresis	1
Vesicoureteral reflux	1
Detrusor overactivity	1
Bladder wall Irregularity	1
Preoperative abnormal findings in Orthopedics	
Lower limb deformity	1
Lower limb movement disorder	1
Lower limb sensory impairment	0
Lower limb abnormal tendon reflexes	1
Surgical complications	0
Outcomes	
Urological improvement	2
Orthopedic improvement	0
Data are presented as the unweighted number (percentage) of patients unless otherwise indicated	

These observations indicate a difference between urological improvement and orthopedic improvement. Several pathophysiological mechanisms have been proposed to account for the differential outcomes of urological and orthopedic manifestations. An established hypothesis suggests that spinal cord tethering impairs blood flow to the distal spinal cord, leading to ischemia, metabolic disturbances, and subsequent neurological dysfunction. When untethering is performed at an early stage, restoration of blood flow and normalization of metabolic activity can occur, thereby enabling, at least in part, reversible functional recovery. This mechanism provides a plausible explanation for the relatively favorable improvement in urological abnormalities after surgical intervention. In addition, it has been argued that bladder and urinary function demonstrate a higher potential for recovery due to the inherent plasticity of neural circuits within the brain-spinal cord axis. The micturition reflex pathway may regain function through reorganization and compensatory neuroplastic changes, allowing patients to experience significant symptomatic improvement even after prolonged dysfunction. Conversely, orthopedic abnormalities, such as scoliosis, joint contractures, and musculoskeletal deformities, tend to reflect chronic and structural changes that have developed over time. Longstanding muscle atrophy, tendon shortening, and bony or spinal deformities often result in fixed lesions with limited reversibility. In such cases, surgical detethering alone is insufficient to restore function, as the underlying musculoskeletal alterations represent structural rather than purely functional pathologies. This pathophysiological perspective highlights why orthopedic manifestations remain refractory to surgical treatment, in contrast to the more favorable trajectory observed for urological symptoms.

These considerations may support the importance of early surgical intervention before irreversible musculoskeletal changes occur and the need for a multidisciplinary approach, including orthopedic management and rehabilitation, to achieve optimal functional outcomes in patients with OTCS.

## Discussion

Recognition of OTCS has fueled substantial debate. Proponents argue that surgical release of the filum terminale in carefully selected patients can lead to clinical and functional improvement [4], whereas critics highlight the risks of surgical intervention in patients lacking definitive anatomical tethering. Recent studies have reported inconsistent outcomes, particularly regarding bladder function [5], leading to calls for prospective trials and standardized protocols [6].

Clinical presentation

Urinary dysfunction, particularly neurogenic bladder, is the most frequently reported OTC feature. In children, it often manifests as urinary frequency, urgency, incontinence, or recurrent urinary tract infections. Even in the absence of overt anatomical abnormalities, urodynamic testing commonly reveals detrusor overactivity, impaired compliance, and sphincter dyssynergia. Multiple clinical studies have emphasized that bladder dysfunction is the most sensitive and reproducible indicator of OTCS [7,8]. Orthopedic and musculoskeletal manifestations are common. Patients may develop scoliosis, lower-limb deformities, pes cavus, or gait disturbances. These findings are thought to reflect a chronic, subtle traction of the spinal cord that interferes with the segmental innervation of the lower limb musculature. Pain radiating to the lumbosacral region or lower extremities may accompany these findings, although pediatric patients generally exhibit such symptoms less consistently than adults.

The neurological signs are often mild or non-specific. Some patients may present with lower-limb weakness, fatigue, or sensory disturbances, although these are typically subtle and may be misattributed to alternative etiologies. Delays in motor development can be observed during early childhood. Reflex changes are rare, and objective neurological deficits are usually less pronounced than those in classic TCS [9].

The natural course of OTCS is typically insidious and progressive, with symptoms emerging in early childhood and gradually worsening during growth. Importantly, symptom severity does not correlate with conventional radiological findings [10].

In summary, the clinical spectrum of OTCS consists of a triad of urological, orthopedic, and neurological findings, with bladder dysfunction being the most reliable clinical indicator. Recognition of these subtle yet functionally significant symptoms is crucial for timely intervention, although the non-specific nature of the findings continues to complicate both diagnosis and surgical decision-making.

Diagnosis

The diagnosis of OTCS remains challenging. Patients present with symptoms consistent with spinal cord tethering despite the absence of radiological features such as a low-lying conus or thickened filum terminale. Consequently, diagnosis requires a combination of clinical evaluation, functional testing, and exclusion of alternative etiologies. MRI is typically used as an initial diagnostic modality to exclude classic TCS, lipomas, dermal sinuses, or other segmental anomalies. In OTCS, the conus usually terminates at a normal or high level, and the filum often appears morphologically unremarkable [2]. All findings are confirmed by two or more board-certified pediatric neurosurgeons, and measurements of the spinal conus medullaris and filum terminale are performed strictly. Subtle findings such as mild thickening or fatty infiltration have occasionally been reported; however, these lack diagnostic specificity and consistency, and functional testing, particularly urodynamic studies, plays a critical role. Multiple investigators have demonstrated abnormalities such as impaired bladder compliance, detrusor overactivity, and voiding dysfunction in patients with OTCS with otherwise normal imaging findings [7,8]. When correlated with urinary symptoms, these findings provide supportive evidence for spinal tethering. Electromyography of the pelvic floor musculature and somatosensory evoked potentials may also be used as adjuncts, although their sensitivity and specificity remain limited. Histopathological evaluation of resected filum specimens has revealed fibrous or glial hypertrophy, vascular hyalinization, and fatty infiltration in some patients with OTCS, suggesting a structural basis for abnormal tension [3]. However, these findings are retrospective and not available preoperatively because no universally accepted definition exists, and diagnostic criteria vary across institutions. Some authors have proposed restricting the diagnosis to cases with characteristic symptoms, supportive functional findings, and symptomatic improvement following filum sectioning. Others caution against overdiagnosis and unnecessary surgery and warn against the liberal use of the term.

Thus, OTCS diagnosis is essentially clinical, functional, and supported but not defined by imaging or ancillary studies. A high index of suspicion is required, and a diagnosis should be made within the context of multidisciplinary evaluation and careful exclusion of alternative causes [11].

Management

Treatment options include surgical intervention with sectioning of the filum terminale and conservative management with symptomatic therapy. Surgical intervention has been advocated on the premise that even subtle traction of the spinal cord can cause neurological and urological dysfunction, regardless of demonstrable radiological tethering. Early reports documented symptom improvement following filum section, particularly regarding bladder dysfunction and orthopedic deformities. Wehby et al. and Valentini et al. reported favorable outcomes in pediatric cohorts with stabilization or improvement of urinary control and motor function [8,11].

More recently, Steinbok et al. conducted a randomized controlled pilot trial demonstrating that filum section produced a measurable improvement in urinary incontinence compared with observation [7]. This study represents one of the few prospective controlled investigations in the field, providing partial evidence in support of surgical intervention; however, other analyses highlight the variability in outcomes and concerns regarding surgical risks. Reported complications include cerebrospinal fluid leakage, wound infection, and retethering. Critics argue that symptom improvement may reflect placebo effects or natural variability in urological symptoms rather than the direct consequences of detethering [10]. Recent reviews emphasize the need for cautious patient selection, restricting surgery to those with progressive, functionally significant symptoms and supportive urodynamic findings [6]. Conservative treatment consists of careful clinical and urological monitoring, physical therapy, and symptomatic management of bladder dysfunction. This approach is often preferred in patients with mild or stable symptoms. Advocates highlight the lack of definitive long-term evidence confirming durable surgical benefits and underscore the importance of avoiding unnecessary interventions in children.

In summary, OTCS management requires an individualized approach. Filum section may relieve symptoms in selected patients; however, the absence of universally accepted diagnostic criteria and high-level evidence necessitates a balanced, multidisciplinary strategy. Future multicenter randomized controlled trials are essential to clarify surgical indications, risks, and long-term benefits.

Prognosis and outcomes

The prognosis of OTCS remains controversial as clinical outcomes after surgical or conservative treatment vary widely across case series. Short- to mid-term studies have suggested that surgical sectioning of the filum results in symptom improvement or stabilization in a substantial proportion of patients. The most consistently reported benefit is improvement in urinary function, with up to 60-80% of pediatric patients demonstrating reduced incontinence, improved bladder capacity, or normalization of urodynamic parameters [12,13]. Orthopedic findings, such as scoliosis and foot deformities, tend to stabilize rather than improve, reflecting chronic musculoskeletal changes. Neurological symptoms such as leg pain or weakness show variable recovery, which is heavily influenced by the duration and severity of preoperative deficits.

However, the prospective data are limited. In a randomized pilot study, Steinbok et al. demonstrated a statistically significant improvement in urinary incontinence in the surgical group compared with the observation group, although generalizability was limited by the small sample size and short follow-up [7]. Long-term follow-up studies have suggested the risks of retethering, particularly in younger children, and the possibility of symptom recurrence several years postoperatively [9]. In conservatively managed cases, relatively stable courses are often observed in patients with mild or non-progressive symptoms. However, some patients exhibit progressive bladder dysfunction or orthopedic deformities, highlighting the unpredictability of their natural history.

Overall, the prognosis of OTCS is heterogeneous and uncertain. The most favorable outcomes are achieved when the intervention occurs before the development of irreversible bladder or musculoskeletal damage. However, the absence of reliable prognostic markers complicates decision-making and underscores the need for long-term controlled studies to define the outcomes more precisely.

## Conclusions

OTCS remains a challenging and controversial condition in pediatric and adult neurosurgery. While it shares clinical features with classic TCS, the absence of radiological signs, such as a low-lying conus or thickened filum, complicates both diagnosis and treatment decision-making. This case series also suggests that OTCS is particularly associated with urological disorders and that improvement can be expected through surgical intervention.

However, treatment outcomes remain heterogeneous, and the risks of complications or retethering must be carefully weighed against the potential benefits of intervention. While conservative management may be appropriate for patients with mild and stable presentations, some cases carry a risk of progression. These considerations underscore the need for objective biomarkers that can facilitate accurate clinical assessment. To establish clear treatment guidelines, prospective multicenter studies with standardized definitions and long-term follow-up are required.
